# An essential role for hepatocyte adenosine kinase in regulating fat metabolism and inflammation

**DOI:** 10.46439/signaling.2.029

**Published:** 2024

**Authors:** Jiayu Yu, Juan Zheng, Chaodong Wu

**Affiliations:** 1 Department of Endocrinology, Union Hospital, Tongji Medical College, Huazhong University of Science and Technology, Wuhan, China; 2 Hubei Provincial Clinical Research Center for Diabetes and Metabolic Disorders, Wuhan, China; 3 Department of Nutrition, Texas A&M University, College Station, Texas 77845, USA

**Keywords:** Adenosine kinase, Fat metabolism, Liver inflammation

## Commentary

Metabolic dysfunction-associated steatotic liver disease (MASLD) is comprised of a spectrum of conditions, which is a progressive form of liver disease ranging from simple steatosis to steatohepatitis. The liver regulates fat metabolic homeostasis through *de novo* lipogenesis, fatty acid uptake and oxidation, low-density lipoprotein secretion in hepatocytes. Simple steatosis is a rather benign and prime condition with increases in fat accumulation in hepatocytes. According to the data from numerous epidemical studies, 75% of MASLD patients is usually viewed having no or slow progression [1]. However, the excessive fat accumulation in hepatocytes is considered having increased lipotoxicity in lipid-related metabolic pathways, which can result in mitochondrial dysfunction and oxidative stress and subsequently interact with multiple cell types within the liver to promote proinflammatory activation or enhance proinflammatory responses. Macrophages, including Kupffer cells (KCs, the resident liver macrophages) and monocytes/macrophages recruited from peripheral blood, play a major role in promoting or limiting the progression from MASLD to metabolic dysfunction-associated steatohepatitis (MASH), depending on macrophage activation status. Better understanding the causative mechanisms underlying fat metabolic homeostasis and inflammation in the liver is of particular importance in developing novel prevention and/or therapeutic strategies for managing MASLD.

Adenosine kinase (ADK) is the enzyme that phosphorylates adenosine to generate adenosine monophosphate, thereby critically regulating intracellular levels of adenosine. The study by Li et al. demonstrated compelling evidence to support an essential role for hepatocyte ADK in serving as an obesogenic gene (enzyme) that promotes excessive fat deposition and liver inflammation [2], which are characteristics of MASLD.

## The Direct Effects of Hepatocyte ADK on Fat Metabolism

ADK deficiency resulting in liver injury and abnormal liver function has been described in several cases. In their study, Li et al. examined the effect of hepatocyte ADK on regulating fat metabolism. The first line of evidence showed that ADK expression was significantly increased in liver cells, predominantly hepatocytes, in human subjects with non-alcoholic fatty liver disease (now MASLD) (including MASH) and in a mouse model with diet-induced obesity and MASLD [2]. This indicates a positive association between ADK amount and excessive fat deposition in the liver. Using three strains of novel mouse models upon hepatocyte specific ADK knock-down and/or overexpression, the authors attempted to define a causal role for hepatocyte ADK and showed that hepatocyte ADK disruption significantly decreased liver fat content and alleviated the degrees of diet-induced steatosis whereas ADK overexpression caused excessive hepatic fat deposition [2]. These lines of evidence strongly demonstrates hepatocyte ADK as an obesogenic enzyme to promote fat deposition in the liver.

Considering that maturity-onset hepatocyte ADK deficiency increased ketogenesis, the authors hypothesized that ADK altered liver fat flux largely through fatty acid oxidation. Using hepatocyte RNA sequencing of mice with hepatocyte-specific ADK overexpression (Hep-ADK^Tg^), the authors showed that down-regulated differentially expressed genes (DEGs) were enriched in pathways including PPAR signaling and the fatty acid and triacylglycerol, as well as ketone body metabolism [2]. The expression of genes related to fatty acid oxidation pathway such as PPARα was significantly decreased in hepatocytes from Hep-ADK^Tg^ mice whereas liver amount of PPARα was significantly increased in maturity-onset hepatocyte ADK deficient mice. In cultured hepatocytes from Hep-ADK^Tg^ mice, PPARα overexpression reversed the decrease in the mRNA levels of key genes related to fatty acid oxidation such as carnitine palmitoyl transferase (Cpt1a) and pyruvate dehydrogenase kinase 4 (Pdk4) [2]. These results indicate that ADK upregulated the expression of hepatocyte genes to promote fat accumulation in the liver. Moreover, the pathways enriched in up-regulated DEGs indicated that ADK regulates the expression of PPARα through aberrant hypermethylation of DNA [2]. As supporting evidence, hepatocyte ADK overexpression increased the transcription of genes capable of promoting methylation reactions and methionine cycle such as S-methyltransferase (Bhmt) and methionine adenosyltransferase 2A (Mat2a). The authors related the high levels of S-adenosylmethionine and S-adenosyl homocysteine, as well as the increased levels of 5-methylcytidine (an indicator of global DNA methylation) with the expression of PPARα [2]. In a substantial study, the authors validated hypermethylation of the promoter and exon1 of PPARα, accompanied by significantly decreased expression of PPARα in livers of Hep-ADK^Tg^ mice [2]. Accumulating evidence suggests ADK increases methylation reactions to regulate the expression of downstream target genes in endotheliocytes, myeloid cells, and neurogliocytes, which is independent of adenosine receptor pathways [3]. Consistently, Li and Zheng et al. gained additional evidence supporting the role for hepatocyte ADK in causing increased DNA methylation and decreased PPARα expression in the context of accelerating hepatocyte fat deposition.

## The Indirect Effect of ADK on Liver Inflammation

It is significant that Li and Zheng et al. gained evidence supporting that hepatocyte ADK acts through promoting dysfunctional hepatocyte-macrophage crosstalk to underlie liver inflammation. For instance, maturity-onset hepatocyte ADK deficiency significantly decreased hepatic proinflammatory signaling through phosphorylated JNK1 (Jnk p46) and phosphorylated p65 subunit of NFkB while hepatocyte-specific ADK heterozygous disruption resulted in decreased macrophage aggregations and proinflammatory signaling in mice fed a methionine and choline-deficient diet [2]. As mentioned above, simple fat accumulation in hepatocytes does not lead to inflammation and damage, but the toxic fats cause mitochondrial dysfunction and oxidative stress. The levels of most hepatic bisphosphate and lysophosphatidylcholine species were increased, and tetralin oleoyl cardiolipin levels were decreased in response to hepatocyte ADK overexpression. This contributed to the activation of mitochondrial dysfunction pathways and oxidative stress, thereby generating mitochondrial DNA (mtDNA).

Hepatic high levels of mtDNA act through cyclic guanosine 2’ 5’monophosphate–adenosine monophosphate synthase–stimulator of interferon genes (STING) signaling to stimulate macrophage STING. Considering that ADK overexpression occurred in hepatocytes but not macrophages, the authors speculated that ADK drove excessive lipid deposition in hepatocytes to impair mitochondrial function. The latter, in turn, resulted in increased production of mtDNA, whose release led to activation of the proinflammatory responses of macrophages via STING. As the first line of evidence, there were increases in the expression of STING and F4/80 in the liver of Hep-ADK^Tg^ mice. In addition, the significantly increased expression of proinflammatory cytokines from macrophages treated with conditioned-media from ADK-overexpressing hepatocytes also validated that ADK-driven proinflammatory mediators functioned to promote macrophage activation. Using single-cell RNA sequencing (scRNAseq) analysis of liver non-parenchymal cells, the authors obtained the results indicating that ADK overexpression in hepatocytes promoted the proinflammatory activation of liver macrophages [2]. This finding is complementary to that of the study by Zhang et al., which shows that ablation of myeloid ADK inhibits the proinflammatory activation of macrophages and reduces cardiovascular atherosclerosis [4]. Given this, Li and Zheng et al. elegantly established the link between ADK expression levels and liver inflammation through showing that ADK-driven factors, via paracrine manners, function to indirectly promote the proinflammatory activation of macrophages. Precisely how ADK-driven hepatocyte factors and STING in macrophages mediate the effects of hepatocyte–macrophage crosstalk on promoting liver inflammation needs to be further investigated.

## Crosstalk of the Liver and Extrahepatic Tissues

Since Hep-ADK^Tg^ mice showed systemic metabolic disorders such as marked increases in body weight and fat mass, as well as insulin resistance, the authors examined extrahepatic tissues in response to hepatocyte ADK overexpression. Compared to control mice, Hep-ADK^Tg^ mice displayed increased adipocyte size, macrophage infiltration, and expression of proinflammatory mediators, as well as decreased expression of hormone sensitive lipase in white adipose tissue (WAT). As the main fat storage location, WAT stores free fatty acids from excessive lipid accumulation in hepatocytes following hydrolysis of endogenous fats and uptake of fatty acids. When excessive in its amount, fats disturb WAT functions. Moreover, bidirectional crosstalk along the gut-liver axis controls metabolism and nutrition responses in the gut and the liver, which reciprocally shapes their functions. Intestinal expression of key genes for nutrient absorption in Hep-ADK^Tg^ mice was significantly increased compared with that in controls, demonstrating hepatocyte ADK-driven communications between the liver and the gut. The crosstalk between the liver and extrahepatic tissue provides evidence that MASLD is a systemic disease affecting obesity and metabolic dysregulation. As such, hepatocyte ADK plays an important role in regulating fat distribution and nutrient absorption throughout the whole body.

## The Advantages of the ADK-related Mouse Models

It is worth noting that Hep-ADK^Tg^ mice started to display a phenotype of metabolic dysregulation around 4–5 months of age, but not at a younger age, e.g., 3 months of age. More specifically, while revealing no differences in hepatic fat deposition and liver proinflammatory responses relative to age- and sex-matched WT control mice at 3 months of age, Hep-ADK^Tg^ mice gained more and more fat deposition in hepatocytes and displayed gradually increased severity of liver proinflammatory responses since 4–5 months of age. Given that ADK overexpression in hepatocytes was the initial trigger, MASLD phenotype in Hep-ADK^Tg^ mice was thought to originate from ADK-driven hepatocyte fat deposition. The latter, when exceeding the threshold at which hepatocytes could handle, brought about fat deposition-driven metabolic dysregulation to initiate and exacerbate hepatocyte proinflammatory responses, at least via a mechanism involving fat-driven oxidative stress [2]. Meanwhile, as mentioned above, ADK-driven hepatocyte mediators were shown to function through a paracrine manner to stimulate macrophage STING expression or activation, thereby exacerbating liver inflammation. As such, the study by Li and Zheng et al. demonstrates that ADK overexpression in hepatocytes causes metabolic dysregulation, which nicely represents or reflects the dynamic changes in liver phenotype of human subjects with MASLD.

Unlike mouse models of MASLD induced upon feeding a high-fat diet with or without high amount of fructose/sucrose, Hep-ADK^Tg^ mice displayed MASLD phenotype under a regular chow diet and can be maintained for more than 18 months (unpublished data). Because of this, hepatocyte-originated changes in fat deposition and proinflammatory responses can be assessed in a manner minimizing the influences derived from dietary responses of extrahepatic tissues (e.g., WAT, the intestine, or even the central nervous system) on MASLD phenotype, and more importantly in a dynamic pattern. The latter is of particular importance in MASLD pathogenesis because in the liver the roles played by hepatocytes vs. macrophages in terms of regulating MASLD aspects vary depending on disease stages. In support of this, the study by Xiao et al. showed that hepatocytes exhibited metabolic adaptation during steatosis (nonalcoholic fatty liver) stage whereas a subset of hepatocytes exerted enhancement in signaling pathways promoting proinflammatory responses that contributed to the development of steatohepatitis [5]. Given this, it would be interesting to determine the extent to which ADK-driven hepatocyte proinflammatory responses, but not macrophage-stimulated inflammation, serve as a predominant contributor exacerbating liver inflammation during the late stage of MASLD including liver fibrosis in the future study.

In summary, the study by Li and Zheng et al. provides convincing evidence that supports a critical role for hepatocyte fat deposition in serving as a key contributor to initiate and exacerbate liver inflammation. In addition, ADK-related mouse models are powerful tools for future studies to mechanistically validate hepatocyte-based target(s) from the perspective of developing novel prevention or therapeutic approaches for precisely managing MASLD.
